# The molecular landscape of hereditary ataxia: a single-center study

**DOI:** 10.1007/s00439-025-02744-y

**Published:** 2025-04-10

**Authors:** Elisa Bregant, Elena Betto, Chiara Dal Secco, Jessica Zucco, Federica Baldan, Lorenzo Allegri, Incoronata Renata Lonigro, Flavio Faletra, Lorenzo Verriello, Giuseppe Damante, Catia Mio

**Affiliations:** 1 Institute of Medical Genetics, Azienda Sanitaria Universitaria Friuli Centrale (ASUFC), Udine, Italy; 2 Department of Medicine (DMED), University of Udine, Via Chiusaforte ingresso E, 33100 Udine, Italy; 3 Mauro Baschirotto Institute for Rare Disease (B.I.R.D.), Costozza di Longare, Vicenza, Italy; 4 Neurology Unit, Department of Neurosciences, Azienda Sanitaria Universitaria Friuli Centrale (ASUFC), Udine, Italy

## Abstract

Hereditary ataxia (HA) is a heterogeneous group of complex neurological disorders, which represent a diagnostic challenge due to their diverse phenotypes and genetic etiologies. Next-generation sequencing (NGS) has revolutionized the field of neurogenetics, improving the identification of ataxia-associated genes. Notwithstanding, repeat expansions analysis remains a cornerstone in the diagnostic workflow of these diseases. Here we describe the molecular characterization of a consecutive single-center series of 70 patients with genetically uncharacterized HA. Patients’ samples were analyzed for known HA-associated repeat expansions as first tier and negative ones were analyzed by whole exome sequencing (WES) as second tier. Overall, we identified pathogenic/likely pathogenic variants in 40% (*n* = 28/70) and variants of unknown significance (VUS) in 20% (*n* = 14/70) of cases. In particular, 10 patients (14.3%, *n* = 10/70) presented pathogenic repeat expansions while 18 cases (30%, *n* = 18/60) harbored at least a single nucleotide variant (SNV) or a copy number variant (CNV) in HA or HSP-related genes. WES allowed assessing complex neurological diseases (i.e., leukodystrophies, cerebrotendinous xanthomatosis and atypical xeroderma pigmentosum), which are not usually referred as pure genetic ataxias. Our data suggests that the combined use of repeat expansion analysis and WES, coupled to detailed clinical phenotyping, is able to detect the molecular alteration underpinning ataxia in almost 50% cases, regardless of the hereditary pattern. Indeed, NGS-based tests are fundamental to acknowledge novel HA-associated genes useful to explain the remaining wide fraction of negative tests. Nowadays, this gap is problematic since these patients could not benefit from an etiological diagnosis of their disease that allows prognostic trajectories and prenatal/preimplantation diagnosis.

**Supplementary Information:**

The online version contains supplementary material available at 10.1007/s00439-025-02744-y.

## Introduction

Hereditary ataxia (HA) belongs to a heterogeneous group of rare neurodegenerative disorders with a wide spectrum of phenotypes. Gait abnormalities, disequilibrium, dysarthria, and dysmetria are the most common clinical traits, associated with degeneration of Purkinje cells and/or spinocerebellar connections (Galatolo et al. 2021). The manifestation of the disease usually occurs between 30 and 50 years old, although an early onset (i.e., before 25 years old) could occur. All inheritance patterns have been detected, including autosomal dominant, autosomal recessive and X-linked inheritance (Jayadev and Bird 2013).

Autosomal dominant spinocerebellar ataxia (SCA) is considered the most frequent dominant HAs, with a prevalence of 5.6 cases per 100,000 individuals, with significant geographical and ethnic variations (De Mattei et al. 2024). It includes more than 40 clinical conditions caused by pathological trinucleotide repeat expansions in SCA1, 2, 3, 6, 7, 8, 12, 17 and DRPLA (dentatorubral–pallidoluysian atrophy), penta-nucleotide expansions in SCA10, 31 and 37, and hexa-nucleotide in SCA36 (Sullivan et al. 2019). Autosomal recessive forms of HA are less frequent and include biallelic variations in more than 100 genes. Also in the recessive forms, repeats amplification can occur, as exemplified by Friedreich ataxia (FRDA) (Beaudin et al. 2019).

Although these conditions have been categorized based on transmission patterns and disease-gene correlations, an increasing number of examples of similarities with a number of clinical syndromes are now emerging. Indeed, HA could present overlapping characteristics with other neurological diseases, such as hereditary spastic paraplegia, inherited peripheral neuropathies, epileptic encephalopathies and mitochondrial disorders (Van de Vondel et al. 2024).

Undeniably, the advent of Next-Generation Sequencing (NGS) in clinical and research settings over the past 20 years has revolutionized the field of genetics in neurological disorders, tremendously improving the identification of gene loci associated with ataxia, with now more than 200 ataxia-associated genes identified (Beijer et al. 2024). Indeed, Whole Exome Sequencing (WES) is widely regarded as the current technology of choice for diagnosing monogenic disorders.

The recent discovery of a remarkable number of genes that, when mutated, produced hybrid phenotypes, ranging from a purer ataxia to pure spastic paraparesis or neurological disorders in which ataxia is only part of the clinical picture, complicates the clinical and genetic diagnostic process. To date, despite the revolutionary advance of genetic testing, genetic diagnosis is still missing for about 50% of HA (Parodi et al. 2018; Alvarez-Mora et al. 2023).

Therefore, within the diagnostic journey for patients presenting hereditary cerebellar ataxia, WES is not the first molecular procedure since it is believed that the most common cause of ataxia is polyglutamine repeats expansions (da Graça et al. 2022).

Given these premises, here we describe the molecular characterization of a consecutive single-center series of 70 patients with genetically uncharacterized HA assessed with a two-step workflow leveraging on the analysis of repeat expansions as first tier followed by WES.

## Materials and methods

### Patient and sample collection

This study uses clinical information and biological samples from 70 individuals referred to the Medical Genetics Institute of the Azienda Sanitaria Universitaria Friuli Centrale (ASUFC) of Udine, Italy, from 2006 to 2024. Informed written consent for research was obtained from all patients for use of their samples in genetic studies. This study was approved by the Institutional Review Board (RIF. Prot IRB-DMED: 294/2024).

### Inclusion and exclusion criteria

For patients’ enrollment, a set of inclusion and exclusion criteria have been used.

Inclusion criteria were: (i) age > 18 years old; (ii) ability to give written informed consent, including compliance with the requirements and restrictions listed in the consent form, by the patients or a parent/legal representative; (iii) documented degenerated brain region (by MRI), ataxia or cerebellar ataxia, gait disturbances and movement problems (i.e. difficulty in walking and climbing stairs, spasticity), nystagmus, dysmetria, slurred speech, dysarthria and dysphagia. Exclusion criteria were: (i) inability to give written informed consent and lack of a parent/legal representative; (ii) documented alcohol-related cerebellar dysfunction; (iii) documented traumatic brain injuries (i.e., concussions).

### DNA extraction

Genomic DNA was extracted from peripheral blood samples collected into 10 ml K_2_-EDTA blood collection tubes using the QIAsymphony^®^ SP/AS instrument (Qiagen) according to the manufacturer’s instruction. DNA quantity was estimated using the Qubit™ dsDNA HS Assay Kit on a Qubit 4.0 Fluorometer (Thermo Fisher Scientific).

### Repeat-primed PCR (RP-PCR)

Repeat-primed PCR was performed to assess *CAG* repeats in *ATXN1* (SCA1), *ATXN2* (SCA2), *ATXN3* (SCA3), *CACNA1A* (SCA6), *ATXN7* (SCA7), *PPP2R2B* (SCA12) and *ATN1* (DRPLA); *CAG/TAG* repeats in *ATXN8* (SCA8); [(CAG)_3_ (CAA)_3_ (CAG)_x_ CAA CAG CAA (CAG)_y_ CAA CAG] repeats in *TBP* (SCA17); *ATTCT* repeats in *ATXN10* (SCA10); GGCCTG repeats in *NOP56* (SCA36); *GAA* repeats *FXN* (FRDA). SCA1/2/3/6/7 analysis was performed with the CE-IVD marked SCAs KIT-FL (Experteam S.r.l.) while SCA8/10/12/17/36, DRPLA and FRDA analysis was performed with a custom-based method (validated in house). A primer labeled at 5’ with a fluorescent molecule located upstream of the repeated sequence and a chimeric reverse primer partially overlapping the repeated region were created. Custom primers and thermocycler conditions are available upon request. After amplification, PCR products were denatured, and fragment analysis was performed on a 3500xL Genetic Analyzer (Thermo Fischer Scientific). Amplicon length of separated fragments was calculated relatively using GS500-LIZ molecular weight standard by the GeneMapper software (Thermo Fischer Scientific). After obtaining the peak pattern, number of repeats was calculated.

### Whole exome sequencing and data analysis

Barcoded libraries were generated from 50 ng of DNA per sample as previously described (Zucco et al. 2024). Reads were aligned to human genome build GRCh38/hg38 and both variant calling and annotation were performed with the Varsome Clinical platform (SAPHETOR). A mean coverage ≥ 50X in at least 95% of the target region was considered suitable of the analysis. Variants were called when a position was covered at least 20 times. An alternate allele fraction of 20% (VAF ≥ 20%) and a genotype quality (GQ) score of ≥ 30 were considered suitable for analysis. Variants with frequency < 1% in population-based databases (i.e., gnomAD) were retained for further evaluation. Variants were classified according to the American College of Medical Genetics and Genomics (ACMG) guidelines (Richards et al. 2015; Miller et al. 2023).

The ExomeDepth algorithm included in the Varsome Clinical platform was used for copy number variants (CNVs) calling. At least 10 different samples, coming from the same sequencing batch, having the same gender and not including related individuals, are used to call CNVs. Observed/expected (O/E) number of reads is then calculated. O/E > 0.8 for deletions and < 1.2 for duplications, were filtered out. CNVs were classified according to the ACMG and the Clinical Genome Resource (ClinGen) guidelines.

In this work, we decided to report both unsolicited findings (UFs) and secondary findings (SFs) as incidental findings (IFs). Indeed, unsolicited findings (UFs) are defined as pathogenic/likely pathogenic variants not related to the initial clinical question but that may nonetheless be of medical relevance for the patient; secondary findings (SFs), instead, are deleterious variants not related to the initial clinical question but that are actively looked for (van der Schoot et al. 2022). Contextually, the secondary findings minimum list (ACMG SF v3.2), which include 81 medically actionable disease gene for which preventive measures and/or treatment exist (Miller et al. 2023), has been used to discriminate between UFs and SFs.

### Sanger sequencing

Sanger sequencing was performed as previously described (Mio et al. 2024). Amplification was performed using 50 ng of DNA and Platinum II Hot Start Master Mix (Thermo Fischer Scientific). PCR primer sequences are available on demand. The amplified products were analyzed by direct sequencing using the Big Dye Terminator Cycle Sequencing Kit v3.1 and capillary electrophoresis on a 3500xL Genetic Analyzer (Thermo Fischer Scientific). Electropherograms were visualized with SnapGene viewer (SnapGene).

### Quantitative PCR (qPCR)

qPCR was performed using the PowerUp SYBR Green master mix (ThermoFisher Scientific) in a QuantStudio 3™ PCR System (Applied BioSystems, Foster City, CA, USA). The ∆∆CT method was performed by the QuantStudio Design & Analysis software v1.4 (Applied BioSystems). qPCR primer sequences are available upon request.

### Oxford nanopore technologies (ONT)-based library Preparation and sequencing

Accurate estimation of *FXN-GAA* repeat length was performed using a targeted long-read sequencing through adaptive sampling. Approximately 2 µg of DNA sheared to a target size of 6 kb using Covaris g-TUBE (Covaris) was used to construct sequencing libraries using the Oxford Nanopore Ligation Sequencing kit V14 (Oxford Nanopore Technologies) following the manufacturer’s instructions. Approximately 50 fmol of library was loaded onto an R10.4.1 flow cell on a GridION sequencer. Target regions comprising 0.2% of the whole genome were enriched using the adaptive sampling option to assess *FXN-GAA* repeats. Sequencing was performed for approximately 2–3 days with one additional library loading. Sequences were base called using Guppy v4.3.4 in high-accuracy mode during the run on the GridION and then aligned to GRCh38 using Minimap2 v2.14. Tandem-genotypes v1.3.0 was used to assess the number of *GAA* repeats.

### Statistical analysis

Statistical analysis was performed using GraphPad Prism Software v.5 (GraphPad Software Inc, Boston, MA, USA). Categorical data were expressed as number and percentage. D’Agostino’s K-squared test was used to assess normal distribution of data. Either Mann Whitney U-test or Welch t-test were used to compare variances between groups. Fisher’s exact test was used for group comparison. P value < 0.05 was considered statistically significant.

## Results

**Fig. 1:**
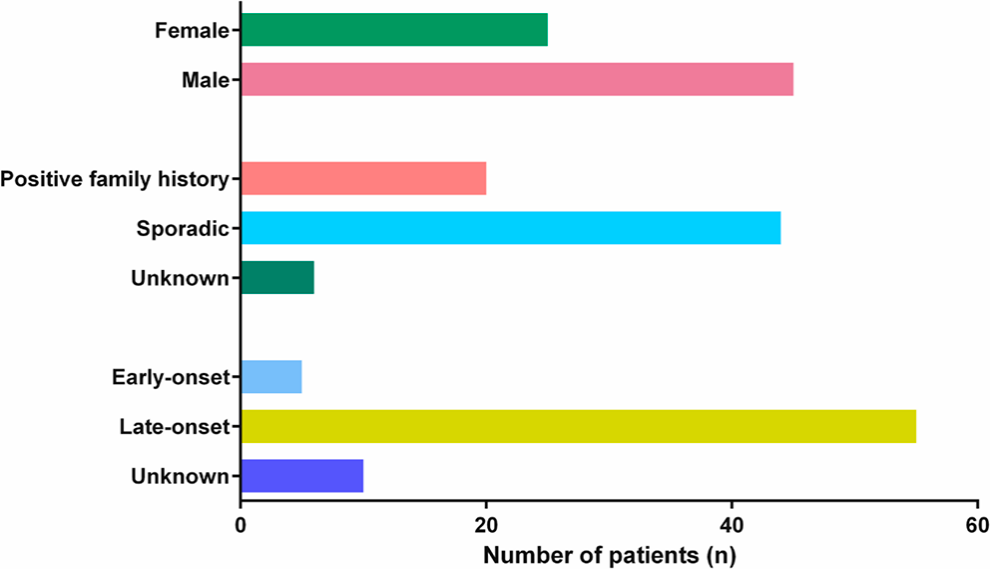
Cohort characteristics

Our cohort includes 70 patients (25 females, 35.7%; 45 males, 64.3%) underwent genetic testing because of a clinical diagnosis of hereditary ataxia/abnormal gait. Samples were collected in an 18-year period (2006–2024). The patients’ median age of was 65.5 years (range: 25–89 years). A positive familial history was found in 28.57% of patients, 62.86% of cases were sporadic and in 8.57% of patients the family history was unknown. Early onset of symptoms was documented in 7.14% of patients, whereas late onset in 78.57% of cases. For 14.29% patients, the age at diagnosis was not available. At least one relative could be assessed in 16 cases for segregation studies and, overall, 26 relatives (7 affected and 19 unaffected) were tested. Cohort-related data are summarized in Fig. 1.

Phenotypically, our cohort was highly heterogeneous. Indeed, patients displaying pure cerebellar ataxia, spastic ataxia and ataxia with peripheral neuropathy were enrolled. Data are summarized in Supplementary Table 1.

In a first analytical setting, patients’ DNA was extracted, and repeat expansion screening was performed to rule out tri-, penta- or hexa-nucleotide expansions known in ataxia-related genes. Overall, 14.3% of samples (*n* = 10/70) bore a pathogenic expansion in either *ATXN1* (*n* = 2/10, 20.0%), *ATXN2* (*n* = 6/10, 60.0%), *TBP* (*n* = 1/10, 10.0%) or *FXN* (*n* = 1/10, 10.0%) (Table 1).

**Table 1:** Pathogenic alteration assessed by RP-PCR

Disease	Phenotype MIM number	Patient ID	Gene	Number of repeats (± 2)
SCA 1	#164,400	HA80	*ATXN1*	29–49 *CAG*
SCA 1	#164,400	HA98	*ATXN1*	30–36 *CAG*
SCA2	#183,090	HA28	*ATXN2*	22–36 *CAG*
SCA2	#183,090	HA35	*ATXN2*	22–37 *CAG*
SCA2	#183,090	HA60	*ATXN2*	24–35 *CAG*
SCA2	#183,090	HA63	*ATXN2*	24–37 *CAG*
SCA2	#183,090	HA71	*ATXN2*	22–37 *CAG*
SCA2	#183,090	HA113	*ATXN2*	22–36 *CAG*
SCA17	#607,136	HA56	*TBP*	38–42 *CAG/CAA ¤*
FRDA	#229,300	HA114	*FXN*	> 100 *GAA*

¤41–48 repeats in TBP are associated to 50% reduced penetrance (Nolte et al. 2010)

To exclude the possibility of *FXN-GAA* expansion in a carrier state and to disclose the exact number of repeats, long-read sequencing on the Oxford Nanopore platform was performed, confirming an estimated biallelic expansion of 124/632 *FXN-GAA* repeats. Patient’s phenotype includes ataxia, dysarthria and spasticity, consistent with the genetic diagnoses of Friedreich Ataxia.

Subsequently, negative samples (*n* = 60) were subjected to singleton-WES to assess a further increase in the diagnostic yield. A positive genetic test was assessed in 30% patients (*n* = 18/60), 5% of which had also an incidental finding (*n* = 3/60), and an inconclusive one in 23.3% (*n* = 14/60), 1.7% of which had also an incidental finding (*n* = 1/60). The detection of solely an incidental finding was highlighted in 6.7% patients (*n* = 4/60). Lastly, 40% of our cohort (*n* = 24/60) tested negative by WES analysis. Autosomal dominant, autosomal recessive and X-linked patterns of inheritance were highlighted.

Overall, 47 variants in 36 patients were highlighted. Specifically, 15 pathogenic (P), 16 likely pathogenic (LP) variants, 14 VUS and 2 disease-associated functional polymorphisms were assessed (Supplementary Fig. 1A). Missense variants were the most common (*n* = 31, 65.96%), followed by nonsense (*n* = 5, 10.64%), frameshift (*n* = 5, 10.64%), splice site variants (*n* = 3, 6.38%), gross deletions (*n* = 2, 4.26%) and in-frame deletions (*n* = 1, 2.13%) (Supplementary Fig. 1B).

Considering positive genetic tests, variants in 20 diverse genes were assessed in 18 patients, indicating a very high level of genetic heterogeneity and a small degree of multiple diagnoses. Genetic and phenotypic data are summarized in Table 2.

**Table 2:** Pathogenic or likely pathogenic alterations HA-related found by WES in our cohort

Patient_ID	Phenotype	Family history	Gene (RefSeq ID)	Variant	ACMG classification	Zygosity	Disease (OMIM ID) Inheritance
HA43	Lower limb weakness, walking difficulties, pyramidal signs, learning disability, muscle atrophy, late-onset cognitive impairment, peripheral motor and sensory neuropathy	Familial	*SPG11* (NM_025137.4)	c.2190dup p.Asp731Ter	Pathogenic	Homozygous	Spastic paraplegia 11 (#604360) Autosomal recessive
HA55	Cerebellar ataxia	Familial	*MFN2* (NM_014874.4)	c.701T > C p.Met234Thr	Likely Pathogenic	Heterozygous	Cerebella ataxia (Elbert et al. 2023) (n.a.) Autosomal dominant
HA58	Neurodegenerative cerebellar ataxia, dysphagia, dysarthria	Sporadic	*TUBA4A* (NM_006000.3)	c.1221G > A p.Trp407Ter	Likely Pathogenic	Heterozygous	Spastic ataxia (Torella et al. 2023; Benkirane et al. 2024) (n.a.) Autosomal dominant
HA61	Lower limb spasticity, ataxic gait, cerebellar atrophy	Sporadic	*SPG7* (NM_003119.4)	c.233T > A p.Leu78Ter	Pathogenic	Homozygous	Spastic paraplegia 7 (#607259) Autosomal recessive
HA72	Cerebellar atrophy, upper limb spasticity, ataxic gait, dysphagia, mild cognitive impairment, fasciculation	Familial	*SQSTM1* (NM_003900.5) *RORA* (NM_134261.3)	c.1175 C > T p.Pro392Leu c.275G > C p.Gly92Ala	Pathogenic Pathogenic	Heterozygous Heterozygous	Frontotemporal dementia and/or amyotrophic lateral sclerosis 3 (Le Ber et al. 2013; Kwok et al. 2014) (#616437) Autosomal dominant Intellectual developmental disorder with or without epilepsy or cerebellar ataxia (Guissart et al. 2018) (#618060) Autosomal dominant
HA74	Microangiopathic leukoencephalopathy, ataxic gait, extrapyramidal signs, rigidity, postural instability, axonal peripheral neuropathy, decreased visual acuity	Sporadic	*MME* (NM_007289.4)	c.467del p.Pro156LeufsTer14	Pathogenic	Heterozygous	Spinocerebellar ataxia 43 (Depondt et al. 2016) (#617018) Autosomal dominant
HA76	Gait difficulties, lower limb muscle atrophy, axonal sensorimotor polyneuropathy	Sporadic	*LMNA* (NM_170707.4)	c.497G > A p.Arg166Gln	Pathogenic	Heterozygous	Muscular dystrophy (#613205) Autosomal dominant
HA88	Ataxia, progressive spastic paraparesis, paroxysmal dyskinesias	Familial	*POLR3A* (NM_007055.4)	c.3028 C > T p.Gln1010Ter c.1909 + 22G > A p.?	Pathogenic Likely Pathogenic	Heterozygous Heterozygous	Leukodystrophy, hypomyelinating, 7 (#607694) Autosomal recessive
HA89	Abnormal gait, dysphagia, dysarthria, memory deficits	Familial	*CPT1C* (NM_001136052.3)	c.109 C > T p.Arg37Cys	Likely Pathogenic	Heterozygous	Spastic paraplegia 73 (#616282) Autosomal dominant
HA90	Episodic ataxia, abnormal gait, paresthesia, dysmetria, downbeat nystagmus	Familial	*CACNA1A* (NM_001127222.2)	c.4737_4742del p.Ile1580_Val1581del	Likely Pathogenic	Heterozygous	Episodic ataxia, type 2 (#108500) Autosomal dominant
HA91	Ataxia	Sporadic	*SETX* (NM_015046.7)	c.482T > G p.Val161Gly c.369_372del p.Leu123PhefsTer37	Likely Pathogenic Pathogenic	Heterozygous Heterozygous	Spinocerebellar ataxia, autosomal recessive, with axonal neuropathy 2 (#606002) Autosomal recessive
HA102	Cerebellar ataxia, abnormal gait, dysphagia, dysarthria, pyramidal signs, sensorimotor polyneuropathy, cognitive impairment, lens opacity	Sporadic	*CYP27A1* (NM_000784.4)	c.1263 + 1G > A p.?	Pathogenic	Homozygous	Cerebrotendinous xanthomatosis (#213700) Autosomal recessive
HA106	Ataxic gait, cerebellar atrophy, unilateral hearing loss, peripheral neuropathy, cognitive impairment	Sporadic	*XPA* (NM_000380.4)	c.772_785del p.Arg258TyrfsTer5	Pathogenic	Homozygous	Xeroderma pigmentosum, group A (Takahashi et al. 2010; Zádori et al. 2020) (#278700) Autosomal recessive
HA115	Ataxia, gait difficulties, dysphagia, psychomotor delay	Familial	*TUBGCP5* (NM_0529053) *CYFIP1* (NM_014608) *NIPA1* (NM_144599) *NIPA2* (NM_001184889)	chr15-22786685-23039553-DEL	Pathogenic	Heterozygous	15q11.2 deletion syndrome (#615656) Autosomal dominant
HA120	Ataxic gait, aphasia, tremor, urinary urgency, progressive cerebellar dysfunction	Sporadic	*GALC* (NM_000153.4)	chr14-87925139-87950754-DEL c.550 C > T p.Arg184Cys ¤ c.1685T > C p.Ile562Thr ¥	Pathogenic Benign Benign	Heterozygous Heterozygous Heterozygous §	Krabbe disease (#245200) Autosomal recessive
HA124	Cerebellar ataxia, nystagmus, diplopia, dysarthria	Unknown	*CACNA1A* (NM_001127222.2)	c.6202 C > T p.Arg2068Ter	Pathogenic	Heterozygous	Episodic ataxia, type 2 (#108500) Autosomal dominant
HA133	Spastic paraplegia, pyramidal signs, ataxia, neurogenic lower urinary tract dysfunction	Sporadic	*ABCD1* (NM_000033.4) *GFAP* (NM_002055.5) *COL4A1* (NM_001845.6)	c.584 A > G p.Gln195Arg c.263G > A p.Arg88His c.904G > A p.Gly302Ser	Likely Pathogenic Likely Pathogenic Likely Pathogenic	Hemizygous Heterozygous Heterozygous	Adrenoleukodystrophy (Qi et al. 2024) (#300100) X-linked recessive Alexander disease (Romano et al. 2024) (#203450) Autosomal dominant Microangiopathy and leukoencephalopathy pontine (#618564) Autosomal dominant
HA139	Cerebellar ataxia, abnormal gait	Familial	*PRKCG* (NM_002739.5)	c.341G > T p.Cys114Phe	Likely Pathogenic	Heterozygous	Spinocerebellar ataxia 14 (#605361) Autosomal dominant

¤ polymorphism; ¥ disease-related functional polymorphism; § *in trans* with *GALC* deletion; not included in Clinvar, Decipher or HGMD professional

Two heterozygous pathogenic variants in *SQSTM1* and *RORA* was detected in patient HA72, a 61 year-old female diagnosed with upper limb spasticity, ataxic gait and mild cognitive impairment. *SQSTM1* variants are associated to frontotemporal dementia and/or amyotrophic lateral sclerosis-3 (FTDALS3, MIM #616437) while *RORA* alterations are associated to intellectual developmental disorder with or without epilepsy or cerebellar ataxia (IDDECA, MIM #618060). The SQSTM1 p.Pro392Leu variant has already been detected in diverse cohorts of patients displaying frontotemporal dementia with amyotrophic lateral sclerosis from different populations (Le Ber et al. 2013; Kwok et al. 2014). It should be noted that the same variant is highly prevalent among patients with Paget’s disease of bone, though with putative incomplete penetrance/variable expressivity (Dessay et al. 2020, 2022). Besides, the same *RORA* variant was detected in an Estonian boy affected by mild cognitive impairment, tremor, cerebellar hypoplasia and ataxia (Guissart et al. 2018). In *vivo* analysis in zebrafish highlighted that the p.Gly92Ala is associated with a dominant toxic effect similar to the one detected in heterozygous *Rora*^*+/−*^ mice, which present a late onset of ataxic gait associated with neuronal loss. Albeit not definitive, the presence of these variants might be consistent with the patient’s phenotype.

In a 48 year-old male (HA102) affected by cerebellar ataxia, pyramidal signs, sensorimotor polyneuropathy, cognitive impairment and lens opacity, a homozygous *CYP27A1* splice site variant was identified. *CYP27A1* alterations are associated with cerebrotendinous xanthomatosis (MIM #213700). This splice site variant has already been highlighted in patients displaying tendinous xanthoma with cataract, neurological and cognitive findings (Jiang et al. 2020; Köroğlu et al. 2024). The patient’s pathognomonic symptoms seem to be consistent with the genetic diagnosis.

A heterozygous pathogenic variant In *XPA* was detected in patient HA106, a 47 year-old male diagnosed with ataxic gait, cerebellar atrophy, unilateral hearing loss and cognitive impairment. Indeed, XPA variants are associated with Xeroderma pigmentosum (XP, MIM #278700). In particular, the p.Arg258TyrfsTer5 variant identified in this patient has been already associated to an unusual XP presentation, with mild cutaneous abnormalities and late-onset neurological impairment (Takahashi et al. 2010; Zádori et al. 2020).

In a 57 year-old male (HA120) affected by ataxic gait, aphasia, tremor, progressive cerebellar dysfunction, three already reported *GALC* variant were assessed. Homozygous or compound heterozygous loss of function variants in *GALC* are associated to Krabbe diseases (KD, MIM #245200), a lysosomal disorder affecting the white matter of the central and peripheral nervous systems with variable age of onset (i.e., childhood, juvenile and adult). To date, a discrete number of pathogenic variants and disease-associated functional polymorphisms have been detected, the latter known to reduce GALC activity by average 10–50% (Shin et al. 2016). Our proband harbored an intragenic ~ 30-kb *GALC* deletion and the known p.Arg184Cys and p.Ile562Thr disease-associated functional polymorphisms. The deletion, spanning from exons 11 to 17, is a common founder variant in the European population and correlates with a loss of the entirety of the smaller 30-kDa subunit and part of the larger 50- to 52-kDa subunit of the GALC protein, resulting in a loss of function (Heller et al. 2023). Both the p.Arg184Cys and the p.Ile562Thr variants, together with the p.Asp248Asn variant, are associated with reduced amount and activity of the mature GALC protein, approximating a hypomorphic variant (Iacono et al. 2022). Indeed, in vitro analysis highlighted how the combination of the p.Ile562Thr with a pathogenic variant (i.e., p.Tyr567Ser or p.Gly286Asp) substantially reduced GALC activity, lysosomal localization and secretion in HEK293 or HeLa cells (Spratley et al. 2016; Shin et al. 2016). Thus, it is postulated that the p.Ile562Thr may function as a genetic modifier (Bascou et al. 2020). The presence of a heterozygous p.Ile562Thr variant *in trans* with a pathogenic variant has been already reported in patients with KD (Saavedra-Matiz et al. 2016) and the occurrence of the p.Arg184Cys polymorphism *in cis* with the 30 kb GALC deletion has been already documented, appearing to be particularly common in KD patients of European ancestry (De Gasperi et al. 1999). Furthermore, Krägeloh-Mann and colleagues described that the combination of p.Arg184Cys and p.Ile562Thr polymorphisms on one allele is found in less than 2% of KD patients and may contribute to KD manifestation if a pathogenic mutation is assessed *in trans*, indeed resembling a hypomorphic allele (Krägeloh-Mann et al. 2017). Indeed, patients with juvenile- or adult-onset KD are typically compound heterozygotes, usually harboring an allele containing a pathogenic *GALC* variant and the other holding a mild missense mutation retaining some GALC activity (Shao et al. 2016; Mächtel et al. 2024). Although we could not perform a segregation analysis, it is possible to predict that the *GALC* deletion and the p.Ile562Thr polymorphisms were inherited *in trans* since the deletion includes exons 9 to 17 while the polymorphisms is located in exon 15. We could not make the same assumption for the p.Arg184Cys variant (located in exon 5) which could be *in cis* with either the deletion or the other polymorphism. Thus, all considered, genetic findings in our proband seem to be consistent with a diagnosis of late-onset KD.

A 55 year-old male (HA133) diagnosed with spastic paraplegia, pyramidal signs, ataxia, neurogenic lower urinary tract dysfunction harbored three deleterious variants associated to three diverse neurological disorders: a hemizygous variant in *ABCD1* associated with adrenoleukodystrophy (MIM #300100); a heterozygous variant in *GFAP* associated with Alexander disease (ALXDRD, MIM #203450); and a heterozygous variant in *COL4A1* associated with microangiopathy and leukoencephalopathy pontine (PADMAL, MIM #618564). *GFAP* variants affecting the p.Arg88 codon have been already highlighted in patients affected by juvenile and adult-onset ALXDRD, showing bulbar signs, ataxia and spasticity as pivotal clinical symptoms (Heshmatzad et al. 2022; Grossi et al. 2024). Notwithstanding, all disorders present abnormal gait/spasticity/ataxia and the age of onset is variable among affected patients. (Romano et al. 2024; Qi et al. 2024). Our patient refused to undergo MRI with contrast medium, so the imaging data obtained so far are not conclusive. Indeed, in patients with adrenomyeloneuropathy, MRI of the spinal cord without contrast shows nonspecific atrophy, whereas abnormalities are evident with gadolinium uptake. Electromyography, however, showed polyneuropathy, slightly worsening, in the lower limbs. The patient’s most recent encephalic MRI (again, without contrast medium) showed no substantial abnormalities. This finding is consistent, as it is known that encephalic MRI can show abnormalities of brainstem pyramidal tracts in about 40–45% of cases. Nevertheless, the patient recently developed pyramidism in the upper limbs. To date, an assessment of the plasmatic concentration of very long-chain fatty acids, useful to either validate or exclude the *ABCD1* variant as causative, has not been performed. The patient’s clinical neurologist, based on the patient’s clinical signs and disease progression, confirmed the diagnosis of adrenomyeloneuropathy (AMN), which is one of the mildest forms of the X-linked form of adrenoleukodystrophy, with age of onset in adulthood. Typically, patients present with lower extremity weakness, difficulty in running, unsteady gait from sensory ataxia, sphincter disorders, and impotence, all of which are present in HA133 patient.

**Fig. 2:**
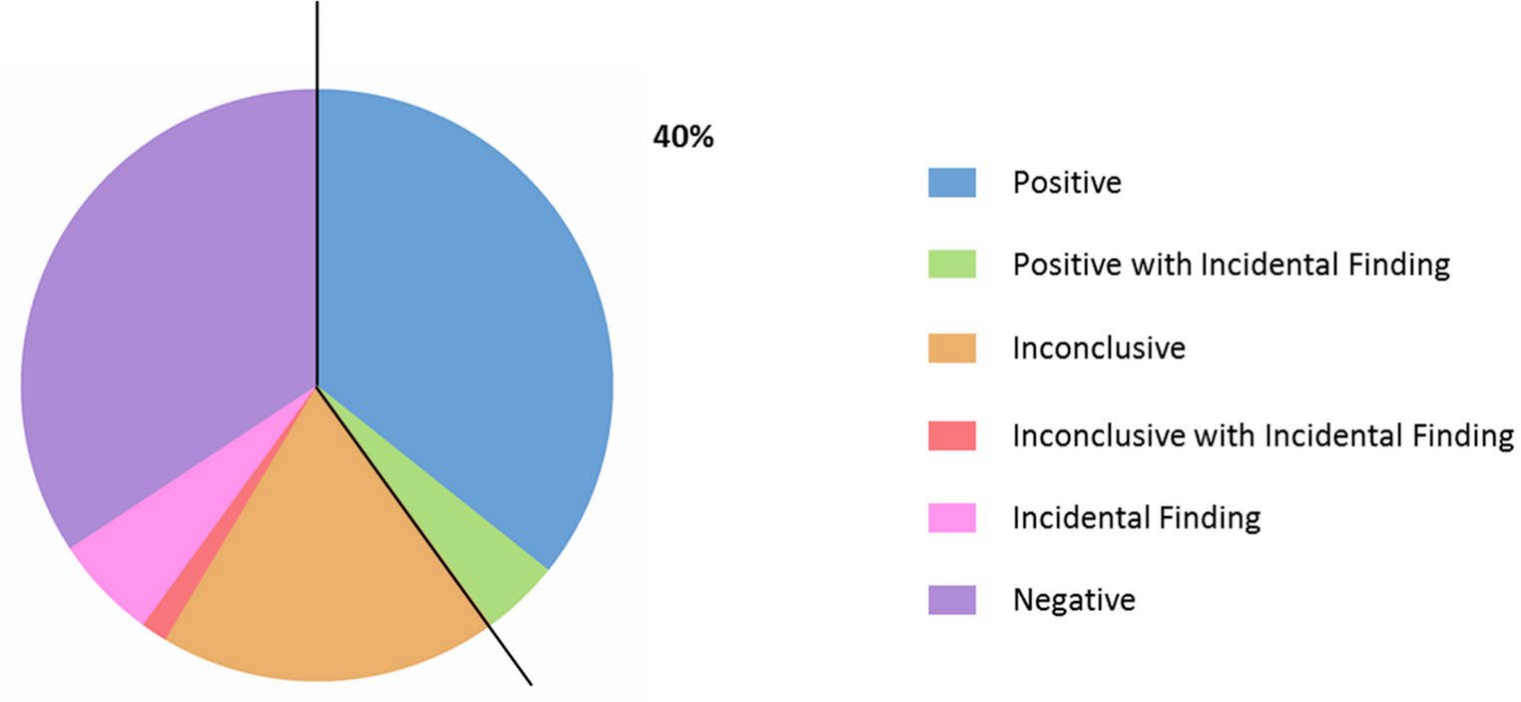
Pie chart showing the overall results of genetic testing. Percentage indicates the number of patients in whom a positive test was assessed. Positive tests are depicted in light blue while positive tests including an incidental finding are represented in green. Negative tests are represented in purple. Inconclusive tests are represented in orange while the concurrent occurrence of VUSs and incidental findings is depicted in red. The solely presence of incidental findings is represented in pink

Overall, considering both repeats expansion and WES data, a positive genetic test was assessed in 40% cases (*n* = 28/70), an inconclusive one in 20% (*n* = 14/70) while the detection of solely an incidental finding was highlighted in 5.7% patients (*n* = 4/70). A negative test was assessed in 34.3% (*n* = 24/70) (Fig. 2). The complete set of molecular data obtained from this analysis, including incidental findings, are summarized in Supplementary Table 1.

Finally, among the 26 patients’ relatives available for segregation analysis, 12 individuals were found to have inherited a deleterious variant (46.2%) and 7 of them were reported to be symptomatic when genetic testing was performed.

## Discussion

Hereditary ataxias represent a diagnostic challenge due to their diverse phenotypes and genetic etiologies, which can manifest at any age and exhibit various modes of inheritance patterns (Tenorio et al. 2023). Hundreds of genes have been associated to HA with heterogeneous pathomechanisms, including tandem repeat expansions, SVs or SNVs in both nuclear and mitochondrial DNA (Sandford and Burmeister 2014).

The discovery of trinucleotide *CAG* repeats expansions as a common cause of HA in the early 1990s boosted the diagnostic pathway of this disease, even in the absence of family history. Nevertheless, the introduction of NGS undoubtedly had a massive impact in expanding the list of genes responsible for complex neurological presentations. NGS has in fact emerged as an effective diagnostic tool for patients tested negative for repeat expansion (Ngo et al. 2020).

Indeed, HA and spastic paraplegia could manifest high degrees of phenotypic overlap also due to the fact that they share alterations in the same genes or in genes involved in the same molecular pathways. Yet that is another reason why NGS, and in particular WES, got a foothold in the diagnostics of these conditions as it is able to assess differential diagnosis within a single test.

In the last 5-years, diverse studies tried to investigate the overall detection rate of NGS in HA, yielding conflicting results ranging from 26 to 52% (Sun et al. 2019; Kang et al. 2019; Kim et al. 2020; Galatolo et al. 2021; da Graça et al. 2022; Gorcenco et al. 2024). Tenorio and colleagues performed a systematic review analyzing 33 primary studies on NGS in ataxic patients assessing a median diagnostic yield of 47.5% (Tenorio et al. 2023). Indeed, the vast majority of collected studies assessed repeat expansions as a first diagnostic step prior to NGS (*N* = 28/33). Thus, more than 50% of HA cases remain unresolved. This gap is problematic because identification of the underlying genetic cause of HA provides patients with an etiological diagnosis of their disease, facilitating prognostic trajectories, prenatal/preimplantation diagnosis and allowing early diagnosis of relatives (Beijer et al. 2024).

In this report, we performed a two-tiered analysis based on repeat expansion assessments and exome sequencing on a cohort of 70 patient with undiagnosed familial and sporadic ataxia. Overall, we identified pathogenic/likely pathogenic variants in 40% (*n* = 28/70) and VUS in 20% (*n* = 14/70) of cases. There was no significance in terms of the number of positive tests between familial and sporadic cases (*n* = 11/20 vs. 17/44, *p* = 0.28). These data diverge from the previously-published literature where a molecular diagnosis was more likely in those with a family history (Bogdanova-Mihaylova et al. 2021). This could be related to the lack of samples from the other affected family members or the unavailability of conducting a trio-based WES analysis. Nevertheless, the age of onset of positive patients is significantly lower compared to the one of patients who received a negative test (*p* = 0.0028).

In detail, 10 patients (14.3%) presented pathogenic repeat expansions while 18 cases (30%) harbored at least a single nucleotide variant (SNV) or a copy number variant (CNV) in HA- or HSP-related genes. As already reported, there is no evidence supporting a higher diagnostic rate for WES compared to RP-PCR (*p* = 0.1383), confirming the fact that the two approaches are not commutable and investigate different pathogenic mechanisms. Furthermore, the age of onset was not significantly different between RP-PCR-positive and WES positive tests (*p* = 0.3096). SCA2 once again proved to be the most frequent autosomal dominant spinocerebellar ataxia in Italy (60% of repeat expansion-positive and 21.43% of positive patients in our cohort) (Brusco et al. 2004; De Mattei et al. 2024).

WES analysis highlighted several rare forms of ataxia, a predictable occurrence given the exponential increase in genes associated with HA. Moreover, it allowed assessing complex neurological diseases, which are not classically counted as pure genetic ataxias. Nonetheless, all patients did have ataxia on clinical examination.

Indeed, the presence of a heterogeneous group of disorders within our cohort but with a clinical diagnosis of ataxia clearly reiterates the notion that grouping neurological disorders with combined symptomatology can be challenging, as the most prominent features or symptoms could vary over time or among individuals with the same affected gene.

Besides, an inconclusive test was assessed in 14 patients, for whom a VUS was identified. For most of them, familial segregation could not be performed, hindering the possibility to reclassify these variants. Supplementary Table 2 summarizes the in silico evaluation of these variants.

We are aware that our study has some limitations. Our cohort included a relatively small number of participants, but truly representative of a real-life scenario. More importantly, our repeat expansion analysis did not include two of the most common repeat expansion disorders in European ancestry patients. Indeed, biallelic penta-nucleotide expansions in in the second intron of *RFC1* are associated to cerebellar ataxia, neuropathy, and vestibular areflexia syndrome (CANVAS, MIM #614575), a frequent late-onset idiopathic ataxia and sensory neuropathy (Ibañez et al. 2024). Since the initial description of *AAGGG* expansions at this locus, several further pathogenic repeat expansion conformations have been described, i.e., *ACAGG*, *AAGGC*, *AGGGC*, and *AGAGG* with slightly different frequencies upon ethnicity (Maltby et al. 2024). Furthermore, more recently, *GAA* expansions in the first intron of *FGF14* have been associated with spinocerebellar ataxia 27B (SCA27B, MIM #620174), a new form of autosomal-dominant SCA representing one of the most common genetic causes of adult-onset ataxia in several populations (Pellerin et al. 2024; Satolli et al. 2024). It is possible that some of the subjects who were negative in the analyses performed in this study or who were carriers of a VUS, could test positive for these types of expansions. Besides, despite neurogenomics has been boosted with rapid advancements in the last decade thanks to NGS, this technology still possesses some weaknesses. Short-read WES sequencing, the approach used in this study, does not detect well long-range structural variants. The use of whole genome sequencing (WGS), which enables the analysis of structural variants (SVs) and non-coding variants (i.e., deep intronic, alteration in promoter/enhancer regions), will allow for the diagnosis of an increased number of positive cases, which however would not contribute much overall to the missing heredity of HA. Long-read sequencing (i.e., ONT or PacBio) has the potential to address some of these limitations, however, this is still an emerging technology and has limitations, including affordability, scalability and technical issues (Rafehi et al. 2023).

## Conclusion

Our data suggest that the combined use of repeat expansion analysis as first tier testing coupled to WES as a second tier test is able to detect the molecular alteration underpinning ataxia in almost 50% cases, regardless of the hereditary pattern. Detailed clinical phenotyping and segregation analysis aid in results interpretations and prioritization of variants during WES analysis. Indeed, WES proved to be able to assess unusual or rare syndromic disorders where the main symptom is ataxia. Moreover, it allowed to confirm that a variety of hereditary diseases overlap the neurological phenotypes or even share genes with HA. Our data, also, reaffirm the extensive genetic heterogeneity underpinning HA, since variants in 20 different genes were assessed in 18 patients.

In conclusion, NGS-based tests are indeed fundamental to dissolve the border between diagnostics and research identifying novel disease-associated genes, expanding the molecular landscape of neurogenetic disorders.

## Electronic supplementary material

Below is the link to the electronic supplementary material.

Supplementary Material 1

Supplementary Material 2

Supplementary Material 3

Supplementary Material 4

## Data Availability

No datasets were generated or analysed during the current study.
