# Supplementary material for: The molecular landscape of hereditary ataxia: a single-center study
Source: Hum Genet. 2025 Apr 10;144(5):545–57. doi: 10.1007/s00439-025-02744-y (PMC12033174; doi:10.1007/s00439-025-02744-y)
Supplement: Supplementary file 1 — Supplementary Material 1 [file 439_2025_2744_MOESM1_ESM.docx]

**Supplementary Figure Legends**

**Supplementary Figure 1**

**Distribution of variants assessed by WES.** Alterations are grouped in terms of (A) ACMG classification and (B) alteration type.
