# Supplementary figures and images for: The molecular landscape of hereditary ataxia: a single-center study
Source: Hum Genet. 2025 Apr 10;144(5):545–57. doi: 10.1007/s00439-025-02744-y (PMC12033174; doi:10.1007/s00439-025-02744-y)

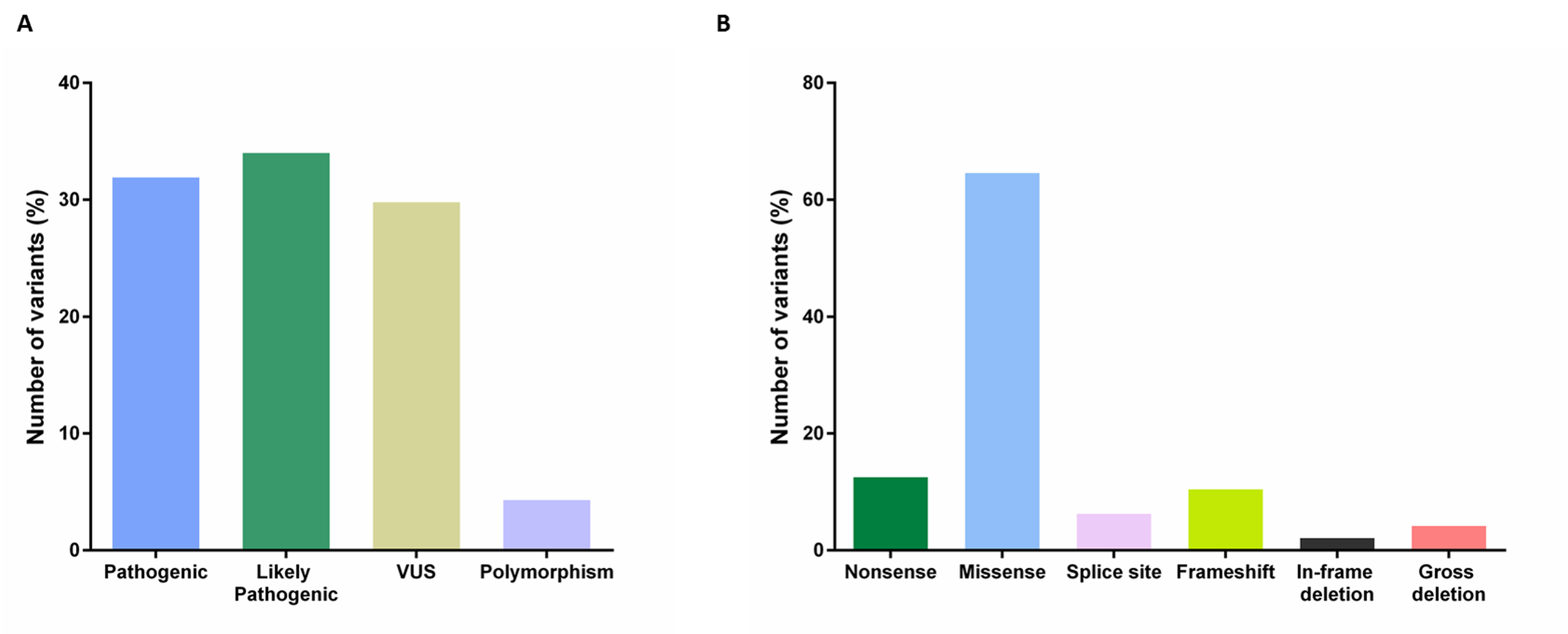

Supplement: Supplementary file 2 — Supplementary Material 2 [file 439_2025_2744_MOESM2_ESM.tif]
